# Pennsylvania Hospital—Clinic of Dr. J. M. DaCosta

**Published:** 1878-03

**Authors:** 


### Selections.

Pennsylvania Hospital.—Clinic of Dr. J. M. Da Costa.—*Salicylic Acid in Acute Rheumatism.*—The patient is twenty-two years of age, and a widow. She inherits the rheumatic diathesis from her father. Last Christmas she took cold, and was confined to bed with chills and fever for a week. Her knees at that time began to swell, and grew red and painful. From that day until now the rheumatism has steadily progressed, involving joint after joint, until it has finally attacked the fingers. The case has plainly been one of an essentially migratory character. First one joint would be affected, then in a day or two the pain and swelling would leave that joint and settle upon another. So the disease has gone on from bad to worse, until, after a partial recovery, followed by a dangerous relapse, the patient was admitted to the wards on February 5th. Her temperature was 101°, with a slight falling remission of less than 1°. The akle-joints, the left in particular, were very much swollen, looking as if they had borne the brunt of successive attacks.

Soon after admission, ten grains of salicylic acid were administered, and the dose repeated every hour until six doses had been taken. The amount of the dose was then reduced so that about a drachm of the acid would be taken in twenty-four hours. The results of this treatment were most striking. On February 8, three days after admission, the temperature fell to 99½°; on the following morning, it was down as low as 99°. This represents the record of last night and this morning.

There has also been a very marked change in the condition of the woman's skin. Upon admission it was harsh; now the skin is soft and the perspiration gentle. The reduction in the amount of pain has been very great. To-day, in fact, the woman is almost free from pain, and can move all her joints except the ankle-joints, without giving rise to any pain whatsoever. The swelling in her hand has wholly disappeared, except in the first metacarpal joints, where there is still some slight stiffness and swelling. The left ankle is also still sore.

After all, however, I am not inclined to lay much stress upon the disappearance of all the external signs of the attack. The internal symptoms of acute rheumatism are always the most dangerous ones. Upon admission the patient exhibited, upon careful auscultation, a slight pericardial friction-sound. This morning I examined her chest again, and although still present, the sound was very slight. One of the good results of our treatment has therefore been that the pericarditis has not been followed by an effusion. There are at present no valvular sounds. I ought to say, just here, that enough morphia was administered in conjunction with the salicylic acid to quiet the patient and ease the paroxysms of pain. The pericarditis was checked by the application of a blister over the heart on February 6, and by subsequent poulticing of the blistered part.

Before sending this case out, I wish to call your attention to four or five points connected with it. Let us (1) examine the state of the left foot and ankle. They are much swollen, and the swelling is not confined to the joint, but has involved all the tissues of the foot. This condition of things is an unusual one, being but rarely found in acute rheumatism. Here the successive attacks, settling on the same joint, have brought on a persistent, inflammatory thickening. This condition will, I fear, result in a permanent stiffening of the joint, a partial anchylosis, such as often succeeds rheumatic gout. We may have an abscess formed. To sum up, we may have certain marked local changes taking place which are unusual in rheumatism, except as sequences of successive localized and unrelieved attacks. There is generally no permanent lesion of the joints or of the connective tissue. Here I fear an exception to the general rule.

I would call your attention (2) to the wonderful effects of the acid in this instance. There had been frequent relapses, and all other treatment had failed, and yet all the symptoms were entirely controlled in three days by the new remedy. You should remember that, as a general rule, if salicylic acid acts at all, it acts promptly. Therefore, salicylic acid, or the salicylate of sodium, to be successful, must be promptly so. If it does not cause a change for the better in the course of two or three days, give it up and try something else. If the acid had shown no good results by to-day in the case of this woman, it would have been useless to continue the treatment longer.

There is a great disadvantage (3) in the administration of large doses of salicylic acid. They may bring on fatal depression. Less than a drachm of the acid in the course of the first twenty-four hours is useless. You ought to be able to administer as much as a drachm and a half without producing any bad results. I prefer the salicylate of sodium to the uncombined acid; it is better borne by the stomach, and can be given in larger doses. I repeat what I said before: there is danger of great prostration following the use of large doses of the acid; therefore, if the pulse becomes feeble and the patient delirious at any time, stop the remedy instantly. Deaths have been reported as following this very prostration and delirium. Another thing: never give salicylic acid in cases of cerebral rheumatism. It is prone of itself to produce dangerous cerebral symptoms.

What (4) about the pericarditis? Has the salicylic acid any influence on pericarditis and endocarditis? What change of treatment does their presence indicate? We must have definite opinions upon these subjects. Salicylic acid has no effect whatsoever upon the cardiac complications of acute rheumatism. Over the fever and pain and swelling it exerts, as you have seen, an excellent influence. Upon the pericarditis and endocarditis it has no effect whatsoever. Where these complications exist you had better unite with the acid some other remedy. In this instance a blister was all that was necessary. In more severe cases, give large doses of digitalis, or of acetate of potassium.

What (5) shall be the after-treatment? The rheumatism has been checked by salicylic acid, and the heart affection has yielded to blistering. There still remain the local thickening and inflammation of the ankle-joint. What shall we do for that? How try to prevent the occurrence of partial anchylosis? I shall order a series of small blisters applied. The part shall then be enveloped in warm-water dressings, so as to keep up constant moisture and secretion. Chronic thickening may haply be thus prevented. In the meanwhile, we must not give up the salicylic acid. We will, however, reduce the dose to forty grains in the course of the twenty-four hours. If the woman shows any tendency to relapse, I shall order her placed on quinia. In all cases of this kind, whether my treatment has been by the alkalies, by large doses of the bromides, or by salicylic acid, I have always treated relapses with quinia. To prevent the recurrence of relapse, I will have this patient take twelve grains of quinia daily. As regards diet, she must have milk, eggs, tea and toast, and occasionally oysters and meat. We shall watch carefully the pericarditis, and apply repeated small blisters to the ankle, followed by fomentations.

Before dismissing the subject, I wish to show you this *post-mortem* specimen of acute pericarditis. The girl from whom it was taken was admitted to the hospital in a dying condition. She had extensive pericarditis with effusion, pneumonia of the right side, and pleurisy of both. Under this complication of disorders, she rapidly succumbed. You see that the whole pericardium is covered with thick lymph. Where pericarditis has reached that stage it is next to impossible to check it. I have brought this specimen before you to show you what pericarditis, in its most advanced stage, really is.

*The Hypodermic Injection of Dialyzed Iron in Chlorosis.*

A. L., aged 21 years, single, has a history of hereditary lung trouble. Thus far the girl herself has given no evidence of any pulmonary disease. She has never had malarial fever nor rheumatism. Last spring she began to feel badly. She lost strength and health, and suffered from frequent attacks of palpitation and dyspnœa. On Christmas last the symptoms became worse; her legs and feet began to swell, and she passed more water than normal. At present her appetite is fair, her bowels are regular, and she sleeps moderately well. There has been a total arrest of the menses for the past three months. Her digestion is only fair. There has been no loss of flesh.

This is a typical case of chlorosis. There are several questions, however, which must first be settled before I proceed to tell you of my new plan of treatment. Is (1) the marked anæmia present in this case connected with any organic cause? And (2) is the swelling of the lower extremities due to cardiac disease, or to disease of the blood? The girl's temperature is about normal, with a range of from 98° in the morning to 99° in the evening. You notice how pale her tongue and gums are. The conjunctiva is pearly, and the ears are pale. Examining the heart, I find that it beats very rapidly. This is probably largely due to the excitement of being before the class. Even in the wards, however, it beats rapidly. The heart-sounds are sharply defined. There is no sign of valvular disease. On the right and left side of the base of the heart I hear a soft systolic murmur. There is no enlargement of the heart. The murmur which I hear is undoubtedly a blood-murmur. This murmur is faintly transmitted into the carotids. I can distinguish a very marked "venous hum" in the jugulars. I have never heard this hum so plainly before. The "venous hum" is a sign of extreme anæmia. I find no cause whatsoever of circulatory disturbance. I see only the signs of a change in the condition of the blood. There is no disease of the liver, lungs, spleen or uterus; no organic trouble anywhere. We call cases of this kind by the name of chlorosis. We are unable to find out why the blood is changed. This girl's blood has been examined microscopically. There is no change in the relative proportion of white to red blood-corpuscles. There is, however, a slight deficiency in red corpuscles. This could not be properly called a case of leucocythæmia. It is undoubtedly chlorisis—menstrual disorder, connected with deficiency of the red blood-corpuscles. The palpitation, dyspnœa, and swelling of the feet are symptoms of the deficiency in the red element of the blood, and not the results of any organic disease.

The girl has improved vastly under treatment. She is getting plenty of rest and good food, but she had them both in abundance before she came to us. Her rapid improvement is altogether due, I think, to a new remedy which I am employing in a very novel manner. I refer to the rapid introduction of iron into the girl's system by means of the hypodermic needle. Why has this not been practicable before the present day? Because it has been wellnigh impossible to obtain a non-irritative form of iron for hypodermic use. The tartrate of iron, although one of the mildest forms, is entirely too liable to cause irritation and abscesses. Lately a new preparation of iron, the dialyzed iron, appeared in the market, which, it is claimed, is neutral and non-irritating. It struck me at once that this was just the thing to be used in my proposed hypodermic injection. I have been using this dialyzed iron hypodermically in this case for the past few days, and it has come fully up to its reputation. There have been none of the usual after-effects of iron, such as costiveness and disordered digestion. All these are done away with.

I have been giving daily hypodermic injections of fifteen minims of pure dialyzed iron. The iron was diluted at first, but, experiencing no unpleasant after-effects, the assistant has, for the past day or so, been using the dialyzed iron undiluted. For the last four days the girl has had a daily injection of fifteen minims. The scars marking the spots where the needle has been introduced show no sign whatsoever of inflammatory action. To-day the patient shall have an injection of twenty, to-morrow of twenty-five, and on the next day of thirty grains of pure, undiluted iron. I think we are going to gain in therapeutics by this case. I certainly expect to find a very rapid change for the better in the girl's condition in the course of the next five or six days. I will bring her before you again and report progress on Saturday next. Between now and then I will see that her blood is carefully examined under the microscope by an expert.

[The girl was again brought before the class two weeks afterwards (February 23). She showed the most wonderful improvement. Dr. Da Costa said: "You will remember that when I last brought this case before you, the blood-murmurs were distinct, and that there had been no menstrual flow for the space of three months. The daily injection of thirty drops of the dialyzed iron under the skin of the girl's arm has not caused the least irritation. Her digestion is admirable, and, what is most wonderful of all, she has menstruated during the past week. Her strength is so much better that she wants to go right home. You see how the color is coming back to her lips, gums and tongue. Another evidence of her very marked improvement is the fact that the 'venous hum,' which was so loud and marked two weeks ago, is comparatively distant and faint this morning. I am convinced of the most positive and marked improvement in the case. The temperature is normal and steady. She feels well, her appetite is good, her bowels regular and her headache all gone.

"Now that we have reached a point of such marked improvement, the question arises as to whether we shall continue this treatment by hypodermic injection, or give it up and place the patient on iron by the mouth. I think we may discontinue the hypodermic medication. In place of it, I will order twenty drops of the tincture of the chloride of iron, in water, thrice daily. You will understand that I do this because I consider the case as practically cured.

"Do I think that we should have had such a rapid cure, and one so unattended with constipation and indigestion, if we had given the iron internally? I think not. You see, therefore, how excellent a method that by hypodermic injection is when the stomach will not retain the iron. Where the stomach will retain slight quantities of iron, we might give a little of the drug by the mouth, and the bulk of it hypodermically.

"Knowing how the iron thus introduced has acted here, we might with advantage employ this treatment in cases of pernicious anæmia. I say we ought to retry the use of iron in pernicious anæmia—try its use hypodermically. The only reason, perhaps, that it has thus far failed to do good in that disease has been because of the great digestive disturbances attending its use.

